# Ozone responses in Arabidopsis: beyond stomatal conductance

**DOI:** 10.1093/plphys/kiab097

**Published:** 2021-02-24

**Authors:** Luis O Morales, Alexey Shapiguzov, Omid Safronov, Johanna Leppälä, Lauri Vaahtera, Dmitry Yarmolinsky, Hannes Kollist, Mikael Brosché

**Affiliations:** 1 Organismal and Evolutionary Biology Research Programme, Faculty of Biological and Environmental Sciences, Viikki Plant Science Centre, University of Helsinki, FIN-00014 Helsinki, Finland; 2 School of Science & Technology, The Life Science Center-Biology, Örebro University, SE-70182 Örebro, Sweden; 3 Institute of Plant Physiology, Russian Academy of Sciences, 127276 Moscow, Russia; 4 Department of Ecology and Environmental Sciences, Umeå University, 90187 Umeå, Sweden; 5 Department of Biology, Norwegian University of Science and Technology (NTNU), NO-7491 Trondheim, Norway; 6 Institute of Technology, University of Tartu, 50411 Tartu, Estonia

## Abstract

Tropospheric ozone (O_3_) is a major air pollutant that decreases yield of important crops worldwide. Despite long-lasting research of its negative effects on plants, there are many gaps in our knowledge on how plants respond to O_3_. In this study, we used natural variation in the model plant Arabidopsis (*Arabidopsis thaliana*) to characterize molecular and physiological mechanisms underlying O_3_ sensitivity. A key parameter in models for O_3_ damage is stomatal uptake. Here we show that the extent of O_3_ damage in the sensitive Arabidopsis accession Shahdara (Sha) does not correspond with O_3_ uptake, pointing toward stomata-independent mechanisms for the development of O_3_ damage. We compared tolerant (Col-0) versus sensitive accessions (Sha, Cvi-0) in assays related to photosynthesis, cell death, antioxidants, and transcriptional regulation. Acute O_3_ exposure increased cell death, development of lesions in the leaves, and decreased photosynthesis in sensitive accessions. In both Sha and Cvi-0, O_3_-induced lesions were associated with decreased maximal chlorophyll fluorescence and low quantum yield of electron transfer from Photosystem II to plastoquinone. However, O_3_-induced repression of photosynthesis in these two O_3_-sensitive accessions developed in different ways. We demonstrate that O_3_ sensitivity in Arabidopsis is influenced by genetic diversity given that Sha and Cvi-0 developed accession-specific transcriptional responses to O_3_. Our findings advance the understanding of plant responses to O_3_ and set a framework for future studies to characterize molecular and physiological mechanisms allowing plants to respond to high O_3_ levels in the atmosphere as a result of high air pollution and climate change.

## Teaser

Natural genetic variation reveals that complex transcriptional regulation and inhibition of photosynthesis differentiate ozone sensitivity in Arabidopsis.

## Introduction

Plants are continuously exposed to adverse environmental conditions that impair growth and fitness (Suzuki et al., 2014). Ozone (O_3_) is a phytotoxic air pollutant that reduces the yield of important crops worldwide (Ainsworth et al., 2012). O_3_ enters the plant through stomata and in the apoplast it breaks down into reactive oxygen species (ROS), such as superoxide ($O_{2}^{–}$) and hydrogen peroxide (H_2_O_2_; Ainsworth, 2017; Waszczak et al., 2018). Depending on the O_3_ concentration, sensitive plant species activate cell death programs leading to the development of lesions (Brosché et al., 2010; Langebartels et al., 2002). O_3_ and most abiotic and biotic stresses increase the formation of ROS with potentially deleterious toxic effects on DNA, proteins, lipids, and carbohydrates. However, ROS are not merely damaging molecules, as they also initiate signaling events that help plants acclimate to stress (Jaspers and Kangasjärvi, 2010; Waszczak et al., 2018).

Plants actively produce ROS as signaling molecules to regulate developmental and defense programs (Huang et al., 2019). One of the earliest detectable responses in defense against pathogens and abiotic stresses is increased apoplastic ROS production (often referred to a ROS burst; Shimada et al., 2003; Choudhury et al., 2017; Qi et al., 2017). As treatments with O_3_ allow a controlled delivery of apoplastic ROS to plants without further manipulation, O_3_ is a very useful tool to study general mechanisms of ROS signaling and its role in cell death, defense signaling, and regulation of gene expression (Vainonen and Kangasjärvi, 2015; Xu et al., 2015a). Apoplastic ROS signaling triggered by O_3_ induces large scale changes in gene expression and metabolic profiles (Blomster et al., 2011; Xu et al., 2015a). However, mechanistic understanding of how ROS regulate gene expression is very limited as only few specific components of ROS signaling have been deciphered in plants. Overall, studies with O_3_ can fulfill two goals at the same time: (1) How do plants protect themselves against this air pollutant? and (2) How do plants use ROS to regulate defense signaling?

The large genetic variation between naturally occurring populations/accessions of Arabidopsis (*Arabidopsis thaliana*) provides a unique resource to study the complex mechanisms underlying stress tolerance. Arabidopsis accessions display different O_3_ sensitivity which is largely explained by stomatal conductance regulating O_3_ uptake and cell death in O_3_ sensitive genotypes (Brosché et al., 2010; Xu et al., 2015b). The O_3_ sensitive accession from the Cape Verde islands Cvi-0 (hereafter, Cvi) has constitutively high stomatal conductance and increased O_3_ uptake caused by impaired function of MITOGEN-ACTIVATED PROTEIN KINASE12 (Brosché et al., 2010; Jakobson et al., 2016). Mutant analysis in Arabidopsis showed that O_3_ activates an abscisic acid (ABA) signaling pathway that ultimately leads to stomatal closure through SLOW ANION CHANNEL1 (Merilo et al., 2013). In addition, several Arabidopsis mutants with increased stomatal conductance display O_3_ sensitivity (Overmyer et al., 2008; Hõrak et al., 2016; Sierla et al., 2018). However, O_3_ responses in Arabidopsis are very complex and clearly involve other physiological functions in addition to stomatal opening (Overmyer et al., 2008). Thus, further characterization of O_3_ responses in sensitive Arabidopsis accessions is needed to unravel genetic and molecular mechanisms underlying O_3_ sensitivity in plants.

Shahdara (Sha), an Arabidopsis accession from Tajikistan in Central Asia was identified as highly O_3_ sensitive (Brosché et al., 2010). Sha is also tolerant to drought and salt stress, has low chlorophyll content, and low levels of ABA (Bouchabka et al., 2008; Sharma et al., 2013; Szymańska et al., 2015; Kalladan et al., 2019). Thus, given its O_3_ sensitivity and altered stress responses, Sha is a good candidate to reveal mechanisms of plant O_3_ responses.

A consistent physiological O_3_ response across many plant species and O_3_ doses is a decreased rate of photosynthesis and reduced expression of photosynthesis-related genes (Fiscus et al., 2005; Wittig et al., 2007; Kontunen-Soppela et al., 2010a; Vainonen and Kangasjärvi, 2015). High O_3_ concentrations reduce the abundance of photosynthetic proteins and pigments, which decrease photosynthetic rates, growth, and biomass production (Ainsworth et al., 2012; Ainsworth, 2017). The use of chlorophyll *a* fluorescence (ChlF) measurements has allowed the assessment of photosynthesis under different stress conditions including O_3_ (Baker, 2008; Bussotti et al., 2011). Measured ChlF parameters in several tree species indicated that O_3_ can affect activities of both Photosystems II and I (PSII and PSI, accordingly; Bussotti et al., 2011). However, in most such studies, the spatiotemporal resolution of ChlF analyses was insufficient to gain insight into kinetics and mechanisms of O_3_-induced damage to photosynthesis.

Transcriptional reprogramming is an early response in plants exposed to abiotic and biotic stresses (Atkinson and Erwin, 2012). Transcriptional responses to O_3_ have been studied in several species: Arabidopsis (Blomster et al., 2011; Brosché et al., 2014; Xu et al., 2015a), rice (*Oryza sativa* L; Ashrafuzzaman et al., 2018), silver birch (*Betula pendula* Roth; Kontunen-Soppela et al., 2010b), and Medicago (*Medicago truncatula*; Iyer et al., 2013). Mutant analysis in Arabidopsis identified regulators of O_3_-induced transcriptional responses, including the plant stress hormones ethylene, salicylic acid (SA) and jasmonic acid (Xu et al., 2015a). Furthermore, cell death induced by O_3_ in Arabidopsis requires altered transcriptional programs (Overmyer et al., 2005). Plants use a large number of transcription factors (TFs) to regulate changes in gene expression (Khan et al., 2018; Tian et al., 2019). In relation to O_3_, TFs from the families ETHYLENE RESPONSE FACTORS (ERF) , TGA, and WRKY regulate some aspects of the O_3_ response (Xu et al., 2015a). However, several more unidentified TFs are likely to be involved (Xu et al., 2015a).

In this study, we characterized molecular and physiological mechanisms underlying O_3_ sensitivity and ROS signaling in Arabidopsis. To that end, we designed a series of experiments with Arabidopsis accessions having different O_3_ sensitivities including Col-0 (hereafter, Col) as O_3_ tolerant, and Sha and Cvi as O_3_ sensitive. O_3_ sensitivity was characterized by measuring stomatal conductance, photosynthetic performance, abundance of antioxidants, and changes in gene expression.

## Results

### O_3_ sensitivity in Sha is associated with increased cell death

**Figure 1:**
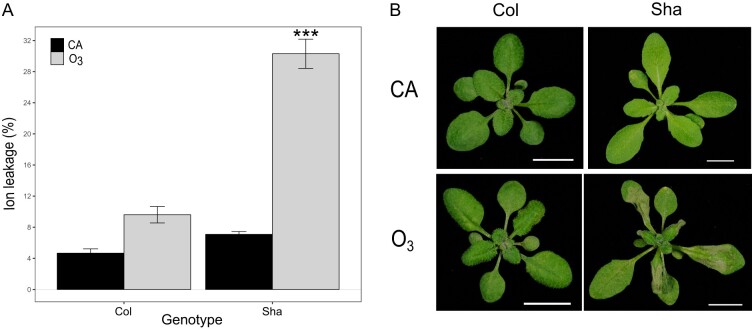
O_3_ response in Col and Sha plants exposed to 350 nL L^−1^ of O_3_ during 6 h. A, Cell death measurements with ion leakage in CA control and O_3_ treated plants. Mean of four independent experiments ± se is shown (*n = *32). The asterisks denote significant differences (*P* < 0.001) between O_3_-induced cell death in Col and Sha assessed with the function fit.contrast from gmodels 2.18.1 (Warnes et al., 2018). B, Representative pictures taken 24 h after the O_3_ exposure was finished (scale bar 1cm).

O_3_ sensitivity in Sha was first characterized by measuring cell death under various O_3_ doses. Exposure to 350 nL L^−1^ of O_3_ for 6 h induced a significantly higher percentage of cell death in Sha than in Col (*P* < 0.001; Figure 1A). Cell death in Sha also corresponded to increased lesion area in the leaves as compared with Col (Figure 1B). Exposure to 200 and 250 nL L^−1^ of O_3_ for 6 h also increased cell death and lesion formation in Sha leaves as compared with Col (*P < *0.05; Supplemental Figure S1). Previous research indicated that Arabidopsis mutants with lower concentration of the antioxidant ascorbic acid (AA) are O_3_ sensitive (Conklin et al., 2000). AA measurements showed that Sha contained approximately 20% lower levels of AA and dehydroascorbic acid than Col both under CA and O_3_ 350 nL L^−1^ for 2 h (Supplemental Figure S2).

### O_3_ sensitivity in Sha was not linked to high stomatal conductance

**Figure 2:**
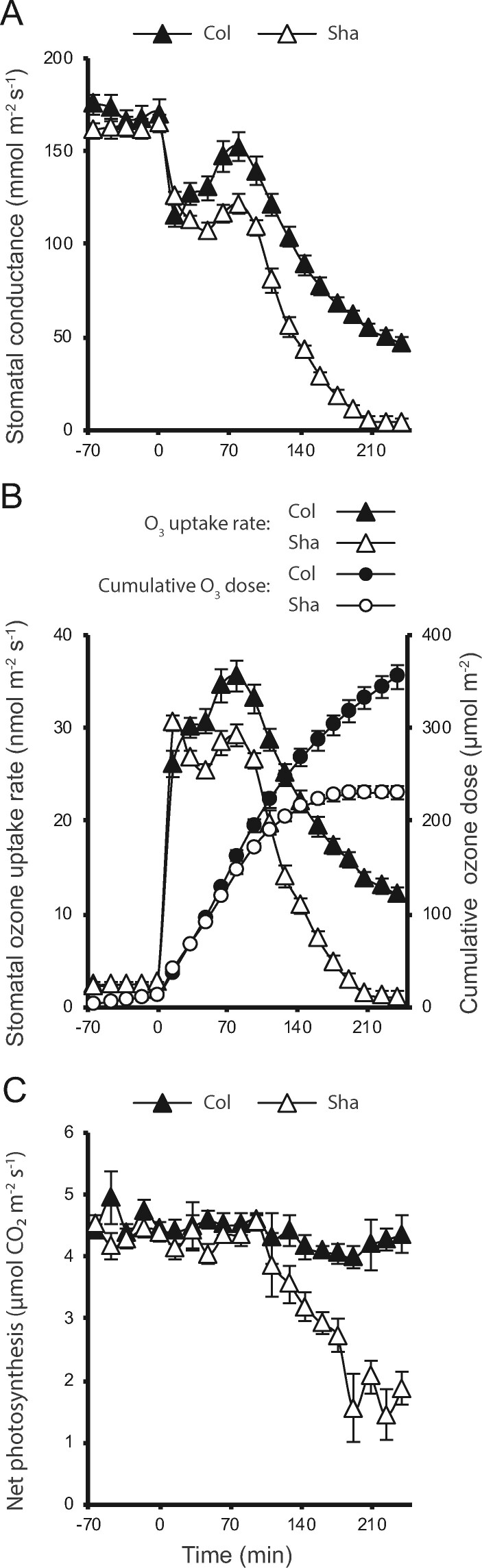
Gas exchange and photosynthetic response in Col and Sha subjected to O_3_ treatments. Time course of (A) stomatal conductance, (B) O_3_ uptake, and (C) and photosynthetic response measured in 3.5-week-old plants exposed to 423 ± 8.2 nL L^−1^ O_3_ during 4 h. Mean of three independent experiments ± se is shown (*n = *12).

Several O_3_-sensitive Arabidopsis accessions display high stomatal conductance and high O_3_ uptake during the first 30 min of acute O_3_ exposure, traits that are positively correlated with O_3_-induced cell death (Brosché et al., 2010). To assess the relationship of gas exchange parameters with the O_3_ sensitivity in Sha, 3 weeks old plants were exposed to O_3_ and stomatal conductance, rate of O_3_ uptake and cumulative O_3_ dose were measured during 4 h (Figure 2, A and B). Col and Sha had similar stomatal conductance in control conditions (Figure 2A, Supplemental Figure S3). In response to O_3_, Col had a rapid drop in stomatal conductance (referred to as rapid transient decrease), followed by reopening of stomata and finally, a sustained decrease in stomatal conductance (Vahisalu et al., 2010). After O_3_ exposure, both Col and Sha showed the same rapid decrease in stomatal conductance (Figure 2A;  Supplemental Figure S3A); however, while Col recovered its stomatal conductance, this response was much weaker in Sha. Although the stomatal uptake rate was slightly different in Col and Sha after 16 and 32 min of O_3_ onset, both genotypes received the same cumulative O_3_ doses during the first 48 min of O_3_ exposure (Figure 2B;  Supplemental Figure S3, B and C). In the continued O_3_ exposure, stomatal conductance in Sha eventually dropped to very low values, while Col still maintained ∼30% of stomatal conductance. Consequently, Sha plants had lower O_3_ uptake and lower total cumulative O_3_ dose as compared with Col plants (Figure 2B;  Supplemental Figure S3, B and C). This indicates that O_3_ sensitivity in Sha is regulated through stomata-independent mechanisms.

### Photosynthesis is severely impaired in Sha by O_3_

To assess the direct O_3_ effects on photosynthetic activity in Sha, we first measured net photosynthesis using gas exchange in three weeks old plants exposed to O_3_ for 4 h. Despite considerably reduced stomatal conductance during O_3_ exposure (Figure 2A), Col maintained its photosynthetic activity. As plants for gas exchange were grown in relatively low light conditions (150 µE), decreased CO_2_ uptake through reduced stomatal apertures was probably not a limitation for photosynthesis (Tanaka et al., 2013). In contrast to Col, net photosynthesis started to progressively decline in Sha approximately 2 h after the onset of the O_3_ treatment (Figure 2C;  Supplemental Figure S3D). Importantly, Col displayed higher net photosynthesis than Sha at approximately the same values of stomatal conductance (256 min after O_3_ onset for Col and 144 min for Sha; Figure 2, A and C). This suggested that the decline of photosynthesis in Sha was not related to stomatal function.

**Figure 3:**
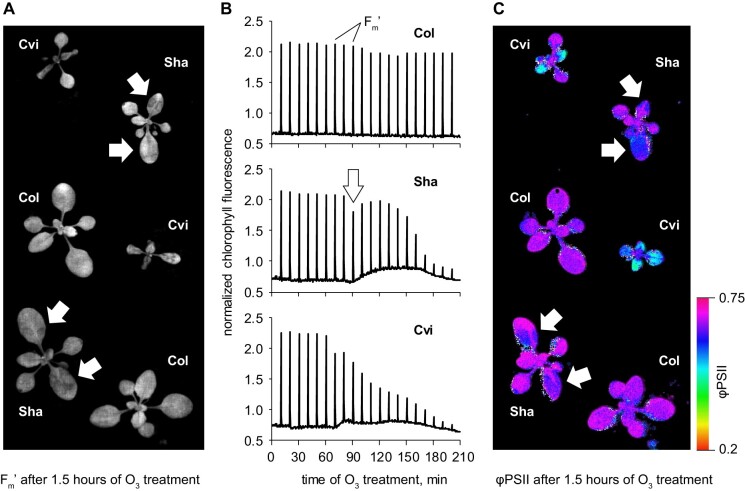
Imaging photosynthesis under O_3_ exposure. A, Representative image of O_3_-induced lesions recorded during a saturating light pulse (*F*_m_ʹ) with the Imaging PAM. Local lesions characteristic of Sha are labeled with arrows. B, ChlF kinetics extracted from the PAM imaging experiment shown in (A). Saturating light pulses to measure *F*_m_ʹ are labeled. Sha-specific transient depression in *F*_m_ʹ is shown with an arrow. C, Effective quantum yield of φPSII in the same experiment as in (A). Arrows indicate local depressions of φPSII in Sha.

In relation to O_3_, photosynthetic traits are usually measured in the whole plant or organ (i.e. leaf) after the specified time of exposure. However, by increasing the spatiotemporal resolution of the measurements, new insights can be gained into what aspects of photosynthesis are the O_3_ targets. We performed real-time monitoring of the O_3_-induced changes of photosynthesis using Pulse Amplitude Modulated (PAM) ChlF imaging. In addition to Sha, we included Cvi as a second O_3_-sensitive accession and compared photosynthetic parameters with those in the O_3_-tolerant Col. Two-week-old plants were exposed to O_3_ and ChlF was monitored from the onset of the O_3_ treatment. Against the background actinic light, saturating light pulses were given every 10 min to image maximal fluorescence, *F*_m_ʹ. After 1.5–2 h of O_3_ exposure, local lesions developed in Sha leaves. These lesions were originally only visible as depressions of *F*_m_ʹ (Figure 3). Notably, the lesions developed in a short time window of 10 min or less, and at the early stage did not coincide with changes in basal light-adapted fluorescence (*F*_s_; white arrows in Figure 3). Quantification of the effective quantum yield of PSII photochemistry (φPSII) revealed difference in photosynthetic electron transfer between the three accessions. No change of φPSII was observed in Col; however, massive drop of φPSII occurred in rosettes of Cvi, while in Sha φPSII originally decreased only within the local lesions (Figure 3C). During the following hour, the Sha lesions expanded, ultimately leading to leaf tissue collapse. This later stage was accompanied by rising *F*_s_, the characteristic feature of disassembling photosynthetic apparatus. In Sha, rising *F*_s_ was accompanied with temporary partial recovery of *F*_m_ʹ, this effect was much less pronounced in Cvi (Figure 3B).

**Figure 4:**
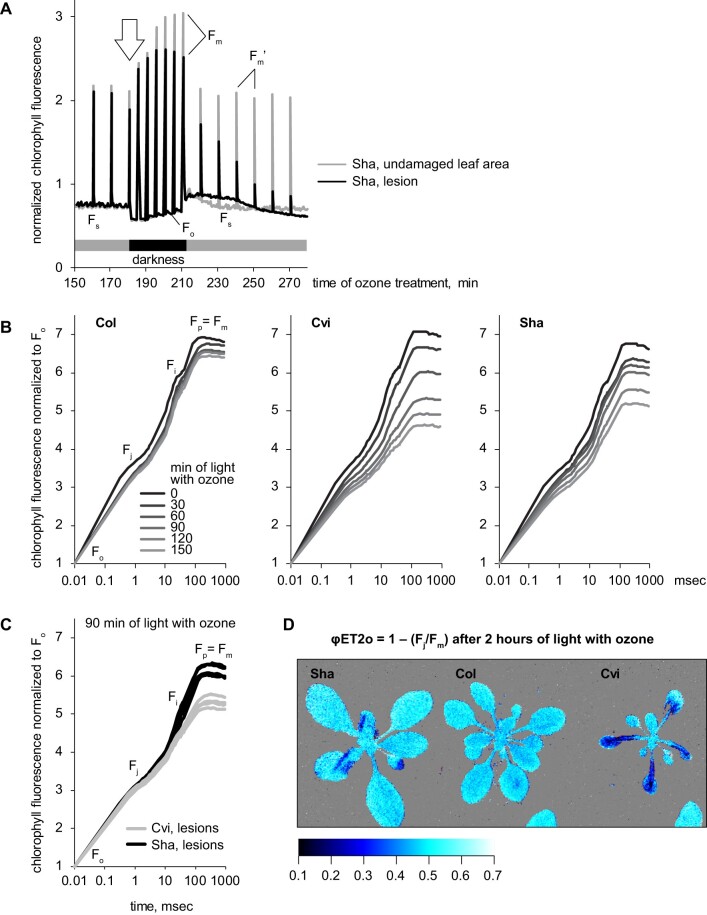
Imaging photosynthesis under O_3_ exposure. A, Kinetics of ChlF extracted after PAM imaging of O_3_-exposed Sha plants. Maximal fluorescence stimulated with the saturating light pulses in light (*F*_m_ʹ) and in darkness (*F*_m_) is indicated. Sha-specific transient drop of *F*_m_ʹ is indicated with an arrow. B, Kinetics of OJIP ChlF rise extracted after OJIP imaging of O_3_-exposed plants. The reads are normalized to *F*_o_. Note the logarithmic time axis. C, Comparison of OJIP kinetics recorded in lesioned tissues of Sha and Cvi after 90 min of O_3_ exposure. Five representative lesions were selected per each accession. The reads are normalized to *F*_o_. D, False-color image of OJIP parameter φET2o associated with electron transfer from PSII to plastoquinone pool.

Quenching of *F*_m_ʹ is referred to as nonphotochemical quenching (NPQ). The two main constituents of NPQ are the energy-dependent quenching (qE) associated with acidification of thylakoids and photoinhibitory quenching (qI) caused by damage to PSII (Baker, 2008). The difference between qE and qI can be revealed by dark adaptation. The qE component dissipates within 10–30 min of darkness, while qI takes longer time to recover. Thus, the PAM imaging protocol was modified to include 30-min dark periods, over which recovery of *F*_m_ was followed with saturating light pulses given once in 5 min (Figure 4A). We selected lesions that had formed just prior to a dark period and extracted kinetics for these areas in all imaged Sha plants (white arrow in Figure 4A). In these lesions, dark recovery of *F*_m_ was incomplete as compared with the undamaged leaf areas. This suggested that the initial drop in *F*_m_ʹ was likely associated with PSII damage, and not with the qE component of NPQ (Figure 4A). Moreover, in the lesioned areas *F*_m_ continued to decline during the dark period, indicating inhibition of PSII activity. The fact that inhibition occurred in darkness hinted that O_3_ exposure triggered programmed light-independent deterioration of photosynthesis.

As a complementary approach, we measured ultra-fast kinetics of ChlF rise (OJIP) in Col, Sha, and Cvi during a 4 h O_3_ exposure (Figure 4B). In essence, this method relies on time-resolved recording of ChlF rise during a saturating light flash. On a logarithmic time axis, this rise reveals inflections *F*_j_ and *F*_i_. The rise of fluorescence from *F*_o_ to *F*_j_ is usually associated with progressive reduction of PSII primary quinone electron acceptor Q_A_. The *F*_j_–*F*_i_ rise is related to reduction of intersystem electron carriers between PSII and Photosystem I (PSI). Finally, the rise from *F*_i_ to *F*_p_ (= *F*_m_) corresponds to reduction of electron acceptors downstream from PSI such as ferredoxin (Bussotti et al., 2011; Stirbet and Govindjee, 2011, 2012). Only small changes in OJIP kinetics were observed in Col, suggesting little effect of the O_3_ treatment on photosynthetic electron transfer. In Cvi, dramatic drop of all OJIP phases was detected over the course of O_3_ exposure, while Sha demonstrated intermediate response. Importantly, both in Cvi and Sha the O_3_-induced decrease in fluorescence was observed as early as at the *F*_o_–*F*_j_ phase (i.e. within 1 ms of OJIP kinetics; Figure 4B). The effects of the qE component of NPQ on OJIP kinetics are known to develop after several hundred milliseconds of illumination (Antal et al., 2011; Shapiguzov et al., 2019). This supported the idea that the O_3_-induced quenching of ChlF was not associated with qE (Figure 4, A and B).

The shape of OJIP kinetics assessed in O_3_ lesions was different between Sha and Cvi (Figure 4C). In Cvi, the decline in *F*_i_–*F*_m_ phase occurred faster than in Sha, while the decline in *F*_o_–*F*_j_ was similar in the two accessions. This suggested that Cvi experienced more rapid changes in electron transfer through PSI, than Sha. The parameter φET2o = 1 – (*F*_j_/*F*_m_) depending on both *F*_j_ and *F*_m_ has been associated with quantum yield of electron transfer from PSII to plastoquinone (Stirbet and Govindjee, 2011; Küpper et al., 2019). O_3_ damage lowered φET2o both in Cvi and in Sha, but the effect was more pronounced in Cvi (Figure 4D). Taken together, these results indicated that the inhibitory effect of O_3_ on photosynthetic functions was mainly associated with PSII damage, and not with the qE component of NPQ. The different OJIP profiles indicate that inhibition of photosynthesis was occurring through different mechanisms in Sha and Cvi. Overall, the measurements of photosynthesis suggested that O_3_ exposure caused programmed decrease of photosynthesis that affected different steps of photosynthetic electron transfer in different accessions.

### O_3_ triggers unique patterns of gene expression in sensitive Arabidopsis accessions

**Figure 5:**
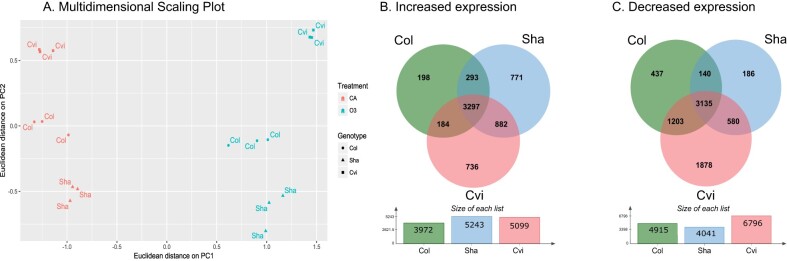
Transcriptional responses induced by O_3_ in Col, Sha, and Cvi. The 3.5-week-old plants were exposed to 350 nL L^−1^ of O_3_ for 2 h, and changes in transcript accumulation were measured with RNAseq (*n* = 3). A, Multidimensional scaling plot of the data, and (B and C) overlap between genes with increased and decreased expression after the O_3_ treatment, respectively.

To gain further insights into mechanisms behind O_3_ sensitivity, we monitored O_3_-induced changes in transcriptome in plants exposed to O_3_ for 2 h with RNAseq. The Sha data were analyzed together with RNAseq data from Col and Cvi with the same O_3_ treatment. Multidimensional scaling plot of the RNAseq data shows clear separation of gene expression patterns detected for the three genotypes (Figure 5A).

The O_3_ effects on transcript levels were determined by performing differential gene expression analysis between clean air (CA) control and O_3_ treatments by genotype. The analysis identified 3,972, 5,243, and 5,099 genes with increased transcript accumulation after 2 h O_3_ exposure in Col, Sha, and Cvi, respectively (FDR ≤ 0.05, log2FC ≥ 1.2; Figure 5B and Supplemental Table S1). Approximately a half of the genes with increased transcript levels were shared between the three accessions. From the other half, almost 78% of the genes were either unique to Sha or Cvi or shared between them (Figure 5B). O_3_ decreased the accumulation of 4,915, 4,041, and 6,796 transcripts in Col, Sha, and Cvi, respectively (FDR ≤ 0.05, log2FC *≤* 1.2, Figure 5C and Supplemental Table S1). Nearly 42% of genes with decreased transcript levels were common between the three accessions. In addition, each genotype had unique genes with decreased transcript levels: 437, 186, and 1,878 genes in Col, Sha, and Cvi, respectively (Figure 5, A and C).

A major rationale for studies in Arabidopsis is that information gained in this model plant should be informative also for other plant species. We used O_3_ transcriptome data from Medicago [70 nL L^−1^, 6 h per day for 6 d (Iyer et al., 2013)) and from rice (108 nL L^−1^, 7 h per day for 8 d (Ashrafuzzaman et al., 2018)) and compared similarities in O_3_ responses between the species. Despite the differences in O_3_ treatments, the expression of Arabidopsis orthologues induced by O_3_ in Medicago and rice had 51% and 67% overlap respectively with Arabidopsis genes (Supplemental Figure S4A). For genes with decreased expression after O_3_, Medicago and rice had 62% overlap with Arabidopsis (Supplemental Figure S4B).

**Figure 6:**
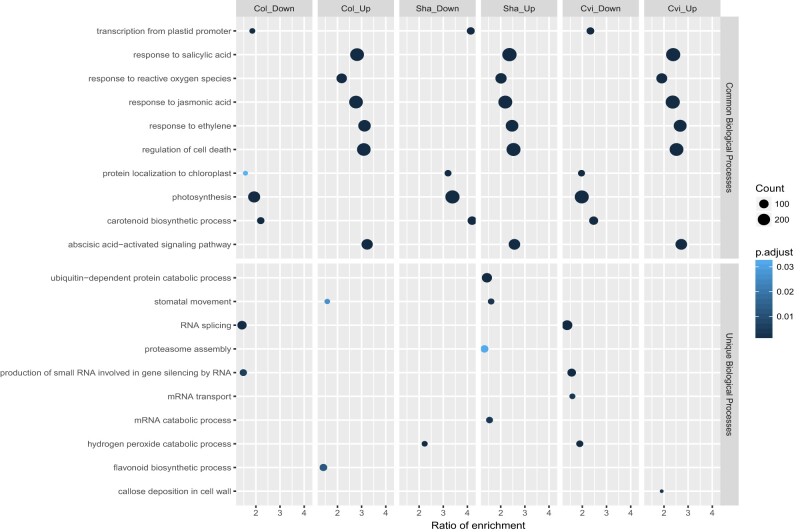
Enrichment analysis of selected GO terms (biological processes) in genes differentially expressed in Col, Sha, and Cvi in response to the O_3_ treatment. Ratio of enrichment (proportion of the total genes annotated to a given GO category that are significantly enriched. Count (number of genes).

We next performed gene ontology (GO) enrichment analysis to get further understanding of the physiological processes regulated by genes differentially expressed by the O_3_ treatment. Figure 6 shows selected common and unique biological processes regulated by O_3_; the complete list of significantly enriched GO terms is provided in Supplemental Table S2. The three accessions shared activation of hormone signaling, for example, response to SA, JA, ethylene, and ABA, regulation of cell death and response to ROS (Figure 6). In relation to chloroplast function, transcript levels decreased for nuclear encoded chloroplast localized proteins, photosynthesis, and carotenoid biosynthesis genes in Col, Sha, and Cvi, respectively (Figure 6). However, the number of genes enriched in these GO categories was higher in Sha than in Cvi and Col. This indicates a greater impact of O_3_ on the expression of photosynthesis-related components in Sha (Figure 6;  Supplemental Table S2).

For genes responding to O_3_ exclusively in Sha and Cvi (Figure 5, B and C), different biological processes were enriched in the two accessions (Figure 6;  Supplemental Table S3). Genes annotated to mRNA and protein catabolic processes, fatty acid, and lipid metabolism among others had increased transcript levels only in Sha (Figure 6;  Supplemental Table S3). The increased transcript levels for flavonoid biosynthesis genes observed in Col was absent in both O_3_-sensitive accessions (Figure 6). In addition, Sha and Cvi had decreased expression levels of genes involved in H_2_O_2_ catabolism. In agreement with differences in stomatal function previously reported for Cvi and Col (Brosché et al., 2010), Cvi displayed misregulation of genes involved in stomata movements that were otherwise induced by O_3_ in Col and Sha (Figure 6;  Supplemental Tables S2 and S3).

Regulation of gene expression in response to stress involves multiple signaling pathways and downstream TFs (Xu et al., 2015a). Large-scale experiments have identified the binding sites of many TFs (O’Malley et al., 2016) and curated databases for TFs and binding sites (TF2Network; Kulkarni et al., 2017). We imported the lists of genes differentially expressed into TF2Network, and identified 729 (Col), 682 (Sha), and 684 (Cvi) TFs as potential regulators of genes with increased transcript abundance under O_3_ (Supplemental Table S4). The three accessions shared 86% of the TFs identified (Supplemental Table S5). Members of the TF families WRKY, ERF, MYB, GATA, and CAMTA, which bind promoter elements of O_3_-responsive genes (Xu et al., 2015a), were detected as regulators of genes induced by O_3_ in Col, Sha, and Cvi (Supplemental Table S5). More than 21% of genes encoding the enriched TFs were themselves induced by O_3_: 155 in Col, 180 in Sha, and 181 in Cvi. Out of these, 22, 25, and 21 O_3_-responsive TFs were distinctively regulated in in Col, Sha, and Cvi, respectively (Supplemental Table S6). Genes encoding regulators of SA signaling (*WRKY38*) and two members of the NAC (for NAM [No Apical Meristem], ATAF1-2 [Arabidopsis thaliana Transcription Activation Factor1-2], and CUC2 [Cup-Shaped Cotyledon2]) TF family (*ANAC04* and *ANAC068*) were highly induced by O_3_ only in Sha (logFC > 3; Supplemental Table S6). The analysis also identified 487 (Col), 417 (Sha), and 480 (Cvi) TFs that bind to promoter elements of genes with decreased transcript accumulation by the O_3_ treatment (Supplemental Table S5). Approximately 75% of these TFs were common between the three accessions indicating similar patterns of gene regulation in response to O_3_ (Supplemental Table S5). Genes encoding the enriched TFs showed also lower transcript accumulation under the O_3_ treatment. Sha had the lowest proportion of TFs downregulated by O_3_ (16.5%) as compared with Col (23.2%) and Cvi (27.2%; Supplemental Table S6).

## Discussion

Natural variation offers possibilities to investigate stress responses that extend beyond those defined with standard laboratory strains. As a model plant, Arabidopsis has been fundamental to understand plant development and stress responses. However, a vast majority of experiments use the accession Col. As Col represents only a limited part of the genetic variation present in Arabidopsis (Alonso-Blanco et al., 2016), the use of additional natural Arabidopsis accessions allows the discovery of mechanisms involved in stress/O_3_ responses. Previously, we associated O_3_ sensitivity in Cvi and other Arabidopsis accessions with more open stomata leading to high O_3_ uptake (Brosché et al., 2010). Similarly, models for predicting plant O_3_ damage rely on O_3_ uptake rates (Fiscus et al., 2005; Mills et al., 2018). In contrast, here we show that O_3_ sensitivity in Sha is not because of increased stomatal conductance or high O_3_ uptake (Figure 2). Hence, in Sha other mechanisms contribute to its O_3_ sensitivity that is independent from stomatal function. Previous research with AA-deficient mutants (Col background; Conklin et al., 2000) revealed O_3_ sensitivity when AA was 1/3 to 1/4 compared with wild-type concentration. It is possible that the lower concentration of AA and dehydroascorbic acid detected in Sha (Supplemental Figure S2) contribute to its O_3_ response. However, it is unlikely that AA is the main determinant of Sha O_3_ sensitivity given the lack of significant effects of the O_3_ treatment on AA levels in both genotypes.

Photosynthesis and chloroplast functions are known O_3_ targets in plants (Clyde Hill and Littlefield, 1969; Fiscus et al., 2005; Bussotti et al., 2011). Our PAM and OJIP measurements revealed that in response to acute O_3_ treatments, photosynthesis was robustly maintained in Col, but decreased in sensitive genotypes, which coincided with development of lesions in the leaves. Both in Sha and in Cvi, O_3_-induced lesions were associated with decreased maximal ChlF in light and darkness (*F*_m_ʹ and *F*_m_, accordingly). This effect has been previously observed, however, in the earlier studies the question whether this was due to the qE or qI component of NPQ, was not fully resolved (Fiscus et al., 2005). Our results indicated that the nature of ChlF decreased in Sha and Cvi was not related to energy-dependent NPQ (Figures 3, 4). OJIP imaging suggested that the damage was associated with altered electron transfer through PSI and with decreased quantum yield of electron transfer from PSII to plastoquinone. Interestingly, repression of photosynthesis developed in different ways in Sha and Cvi. Importantly, O_3_-induced decay of photosynthetic functions continued in darkness. Light-independent PSII damage has previously been associated with heat stress and over-reduction of plastoquinone pool (Marutani et al., 2012). Our results suggest that similar effects may occur in Sha in response to O_3_. In maize (*Zea mays* L), the effect of O_3_ on photosynthesis was dependent on genotype, that is, it is a heritable trait, and improved photosynthesis is a possible target in breeding for O_3_ tolerance (Ainsworth, 2017; Choquette et al., 2019). Our results in Cvi and Sha refine the direct target of O_3_ in photosynthesis and can help design new screens for O_3_ tolerance. Previous studies have also indicated the potential for combining phenotyping methods using ChlF with high-throughput genotyping methods as a promising approach for elucidating the basis for O_3_ tolerance in sensitive crops (Ainsworth et al., 2014). As photosynthesis can be monitored in vivo with high space and time resolution, we propose that our photosynthetic measurements could be useful in large-scale phenotyping and breeding programs.

The O_3_ treatments used in Arabidopsis typically include higher doses and shorter exposure times than those used in crop species; however, even if experiments with Arabidopsis use relatively high levels of O_3_, they are still relevant to understand plant O_3_ responses at lower doses. The O_3_ transcriptional responses determined in this study had >50% overlap with O_3_ regulated genes in Medicago (Iyer et al., 2013) and rice (Ashrafuzzaman et al., 2018; Supplemental Figure S4). Furthermore, mechanisms first identified with high O_3_ treatments in Arabidopsis have been key to understand plant defenses at lower doses in other plant species, for example, the identification of AA-deficient Arabidopsis mutants (Conklin et al., 2000).

In addition to the large overlap in O_3_-regulated transcripts between accessions, we also show accession-specific responses (Figures 5 and 6; Supplemental Tables S1–S6). Our data indicate that impaired regulation of genes involved in flavonoid biosynthesis and ROS metabolism may contribute to O_3_ sensitivity in Sha and Cvi. Furthermore, O_3_ sensitivity in Sha could be mediated by additional mechanisms that involve transcriptional regulation of genes with catalytic functions (Figure 6;  Supplemental Table S2). These differences in gene expression between Sha and Cvi under O_3_ further indicate that O_3_ sensitivity in Arabidopsis is controlled by multiple mechanisms at the level of transcription. One mechanism could involve the activation of different TFs as indicated in our data (Supplemental Table S6). We identified candidate regulators of genes responding in Sha and Cvi, some of them being highly induced by the O_3_ treatment. Future studies exploring the roles of these TFs in O_3_ responses will help to understand O_3_ sensitivity and ROS signaling in plants.

In response to changes in the environment, plants activate signaling pathways to alter transcriptional responses. Application of a chemical that inhibits RNA polymerase II leads to a reduction of O_3_-induced cell death (Overmyer et al., 2005). This directly demonstrates that altered transcription is an important aspect of plant responses to O_3_. The breakdown of O_3_ in the apoplast to various ROS activates the plant enzymatic machinery for further ROS production (Wohlgemuth et al., 2002; Ainsworth, 2017). Active production of apoplastic ROS is triggered by several stresses and is a prominent feature in the defense against pathogens (Qi et al., 2017). Accordingly, there is a large overlap in pathogen- and O_3_-regulated transcriptional changes (Vaahtera et al., 2013; Xu et al., 2015a; Vuorinen et al., 2020). In agreement with these previous studies, we report in the three accessions the O_3_-induced expression of genes involved in defense response to pathogens including fungus and bacteria, to wounding and to several abiotic stresses such as drought, heat and high light (Supplemental Table S2). The ROS burst produced under many stresses could also, at least partially, explain the phenomenon of cross-tolerance, where treatment with one stress confers tolerance to other stresses (Perez and Brown, 2014). For example, pretreatment with O_3_ confers tolerance to virus infection (Sudhakar et al., 2007). Thus, the identification of mechanisms regulating plant O_3_ responses has broad implications for understanding plant defense responses, which go beyond the role of O_3_ as an air pollutant.

In summary, our study reinforces the importance of genetic variation as a tool to unravel molecular mechanisms of plant responses to O_3_. We show that these reactions are complex and mediated by multiple mechanisms, as different O_3_-sensitive accessions display different molecular and physiological responses to O_3_. Furthermore, our data demonstrate that mechanisms independent of stomatal conductance are also key in these processes. Our findings set a framework for future studies aiming at characterizing molecular and physiological mechanisms allowing plants to respond to high O_3_ levels in the atmosphere as a result of high air pollution and climate change.

## Materials and methods

### Plant material and growth conditions

Seeds of the Arabidopsis (*A. thaliana*) accessions Col, Sha, and Cvi were obtained from Nottingham Arabidopsis Stock Center. Seeds of all genotypes used in the experiments were harvested from plants grown under the same conditions. Seeds were sown on 1:1 peat/vermiculite, stratified for 3 d, and then grown at 22/19°C (day/night) for a week. For cell death and transcript accumulation measurements, four geminated seedlings were transplanted into 8 × 8 cm^2^ pots containing fresh 1:1 peat/vermiculite mixture. For photosynthesis measurements, plants were transplanted to a tray containing six pots. Subsequently, plants were grown in controlled environment chambers (Weiss Bio1300; Weiss Gallenkamp) under short day conditions (12/12 h d/night photoperiod) with 250 μmol m^−2^ s^−1^ irradiance at 22°C/18°C (day/night) and 70%/90% relative humidity. All plants were grown under the same conditions until they were used for the experiments. Plants used for gas-exchange experiments were grown as previously described (Kollist et al., 2007).

### O_3_ treatments and cell death measurements

Three-week-old Col and Sha plants were exposed to O_3_ (350–423 nL L^−1^) in parallel with CA controls that consisted of unfiltered ambient air with normal background O_3_ concentrations 10–20 nL L^−1^, which have no effects on plants (Overmyer et al., 2000). The exposure times ranged from 2 to 6 h depending on the measured response.

O_3_-induced cell death was quantified in plants exposed to O_3_ 200–350 nL L^−1^ for 6 h. From five to eight individual rosettes per O_3_-treated and CA controls were harvested and soaked into 12 mL of Milli-Q water for 18 h. Thereafter, electrolyte leakage was measured with a conductivity meter (Model FE30; Mettler Toledo, Germany). The total electrolyte content was measured after freeze–thawing and data are expressed as percentage of total ions. The experiments were repeated four times.

### Stomatal conductance and gas exchange measurements

Steady-state stomatal conductance and photosynthesis rate were measured from Col and Sha plants under controlled conditions with a GFS-3000 gas exchange system (Walz, Effeltrich, Germany) using a whole Arabidopsis rosette cuvette. Stomatal conductance was also measured using a Delta-T Device porometer with a clip-on cuvette (Model AP4; www.delta-t.co.uk). For O_3_-induced stomatal closure and the diurnal stomatal aperture experiments, gas exchange was monitored with a custom-built gas exchange device, and data analyzed as previously described (Kollist et al., 2007).

### Spectroscopic measurements of photosynthesis

Photosynthetic performance was imaged with PAM ChlF imaging (Imaging-PAM, M-series; Heinz Walz, Germany) and a FluorCam FC 800-C/1010 CUST with Fast Camera TOMI-3 (P.S.I., Czech Republic; Küpper et al., 2019; Shapiguzov et al., 2020). Col, Sha, and Cvi seedlings were transplanted 1 week after germination to a tray containing six pots and grown under 220–250 µmol m^−2^ s^−1^ and a 12/12 h d/night photoperiod for a week. The 2- to 3-week-old plants were treated with O_3_ directly inside the imaging devices. Imaging was performed in the morning. For PAM imaging, the minimal (*F*_o_) and maximal (*F*_m_) fluorescences were determined before the lights turned on. Then actinic light (200 µmol m^−2^ s^−1^) was generated by the device light-emitting diode (LED) light sources. O_3_ exposure started 1.5 h after the onset of actinic light. Saturating flashes were triggered every 10 min to assess maximal fluorescence under light (*F*_m_′). The effective quantum yield of PSII photochemistry (φPSII) was calculated as φPSII = (*F*_m_′ – *F*_s_)/*F*_m_′ (Genty et al., 1989). The kinetics of ChlF was normalized to *F*_o_. For the imaging of OJIP (*F*_o_, *F*_j_, *F*_i_, *F*_p_) transients, plants were shifted in the morning from growth light conditions to the imaging system that was pre-equilibrated with O_3_ (350 nL L^−1^). Immediately after the shift, the plants were dark-adapted for 10 min, after which OJIP at time 0 was imaged. Then consecutive 30-min periods of actinic light (200 µmol m^−2^ s^−1^) started, each followed by a 10-min dark adaptation and OJIP imaging. The OJIP imaging protocol included three measurements of the background signal, then three 20-µs flashes of saturating light for *F*_o_ measurement and finally a saturating flash (1.2 s of 3,500 µmol m^−2^ s^−1^). During the saturating flash, images were recorded at 0, 0.3, 0.6, 0.9 … 5.1 ms; 5.4, 7.8, 10.2 … 101, 4 ms; 102, 132, 162 … 1,092 ms following the start of the pulse. Three background and three *F*_o_ values were averaged.

### RNA sequencing

The 3-week-old Col, Sha, and Cvi plants were exposed to O_3_ 350 nL L^−1^ and CA for 2 h. Four rosettes per treatment and genotype were harvested immediately after exposure, snap-frozen in liquid nitrogen and stored at –80°C until analyzed. Total RNA was extracted with TRIzol (Invitrogen). RNA quality was checked with Agilent 2100 Bioanalyzer and the concentration measured with nanodrop ND-1000 (NanoDrop Technologies). RNAseq library preparation and sequencing were performed at the Institute of Biotechnology, University of Helsinki using three biological replicates. Libraries were constructed using TruSeq Standed mRNA Sample PrepKit (Illumina) following manufacturer’s instructions. The library concentration was measured using Qubit Fluorometer, and the quality and size were checked by Fragment Analyzer (Advanced Analytical, AATI). Libraries were sequenced on NextSeq 500 (Illumina).

RNAseq data analysis was done in Chipster (Kallio et al., 2011) and in R (R Development Core Team 2018), version 3.5.0. The quality of raw reads was inspected in Chipster with FastQC (Andrews, 2014). Removal of adapter sequences, trimming and cropping of the reads was done using Trimmomatic-0.33 (Bolger et al., 2014) in single-end mode. The bases with a Phred quality score less than 20 were trimmed from the ends of the reads and reads shorter than 30 bases were removed from the analysis (-phred33, TRAILING:20 and MINLEN:30). Filtered reads were mapped to the Arabidopsis transcript reference database AtRTD2 (Zhang et al., 2017) using Kallisto V-0.43.0 (CMD:quant; Bray et al., 2016) with 4,000 bootstrap sets. The final count table for each biological replicate was obtained as the mean of the bootstrap runs. The count table was used as input to edgeR (v 3.14.0; Robinson et al., 2009) to carry out differential gene expression analysis. Genes with no expression were removed and the filtered count table was normalized using the default Trimmed Mean of *M*-values. The glmLRT method was used to fit the statistical model in edgeR, and Benjamini–Hochberg false-discovery rate correction of *P*-values was used to adjust for multiple testing, with false discovery rate (FDR) ≤0.05 as significance threshold.

The overlap between lists of genes differentially expressed genes by O_3_ was visualized in jvenn (Bardou et al., 2014). Venn diagrams were also used to compare genes induced by acute O_3_ exposure in our study with Arabidopsis orthologues regulated by chronic O_3_ exposure in Medicago (Iyer et al., 2013) and in rice (*O. sativa* L; Ashrafuzzaman et al., 2018). Arabidopsis orthologs from Medicago (*M. truncatula*) were reported in (Iyer et al., 2013) and those from rice were obtained from the Rice Genome Annotation Project (http://rice.plantbiology.msu.edu/home_overview.shtml). GO term enrichment was performed using clusterProfiler (Yu et al., 2012). The ratio of enrichment, that is the proportion of the total genes annotated to a given GO category which are significantly enriched in a particular gene set, was calculated by dividing the clusterProfiler estimated parameters gene ratio by the background ratio.

Genes differentially expressed by the O_3_ treatment were further analyzed by searching for promoter elements in their promoter regions. Enrichment of promoter elements was implemented in TF2Network including 1,793 curated binding site elements corresponding to 916 TFs (Kulkarni et al., 2017).

### AA measurements

The concentrations of total AA and dehydroascorbate were determined spectrophotometrically according to (Gillespie and Ainsworth, 2007). Three-week-old Col and Sha plants were exposed to 350 nL L^−1^ of O_3_ or CA for 2 h. Measurements from fresh leaves were performed immediately after the O_3_ treatment.

### Statistical analysis

Statistical analysis was performed in R. Linear mixed-effects models with replicates as random-grouping factors were fitted and two-way analysis of variance was calculated using function lme from package ‘nlme’ (Pinheiro et al., 2018). Function fit.contrast from package gmodels 2.18.1 ( Warnes et al., 2018) was used to fit pairwise contrasts defined a priori and *P*-values adjusted with the function p.adjust. Figures were plotted using ggplot2 (Wickham, 2009).

### Accession number

RNAseq raw data were deposited at Gene Expression Omnibus with the accession numbers (GSE65740 and GSE117052).

## Supplemental data

The following materials are available in the online version of this article.

**Supplemental Figure S1.** O_3_ response in Col and Sha plants treated with two different doses of O_3_ for 6 h.

**Supplemental Figure S2.** Ascorbic acid measurements in Col and Sha plants exposed to 350 nL L^−1^ O_3_ for 2 h.

**Supplemental Figure S3.** Gas exchange parameters in Col and Sha subjected to O_3_ treatments.

**Supplemental Figure S4.** Identification of common O_3_ regulated genes in Arabidopsis, Medicago, and rice.

**Supplemental Table S1.** List of differentially expressed genes in Col, Sha and Cvi after 2 h O_3_ (350 nL L^−1^) treatment as determined with RNAseq (EdgeR, FDR ≤ 0.05).

**Supplemental Table S2.** List of significantly enriched GO terms associated to differentially expressed genes (FDR ≤ 0.05).

**Supplemental Table S3.** List of significantly enriched GO terms associated to O_3_ regulated genes exclusively in Sha and Cvi (FDR ≤ 0.05).

**Supplemental Table S4.** List of transcription factors whose motifs were significantly enriched in the promoter of differentially expressed genes (FDR ≤ 0.05).

**Supplemental Table S5.** Overlap between TFs predicted to regulate the expression of genes responding to the O_3_ treatment.

**Supplemental Table S6.** Overlap between genes encoding TFs predicted as regulators in the enrichment analysis which were differentially expressed by the O_3_ treatment.

## Supplementary Material

kiab097_Supplementary_Data

## Notes

L.O.M. and M.B conceived the research; L.O.M., A.S., J.L., D.Y., and L.V. performed experiments; L.O.M., A.S., D.Y., O.S., and H.K. performed data analysis; L.O.M. and M.B. wrote the article with contributions of all the authors.

The author responsible for distribution of materials integral to the findings presented in this article in accordance with the policy described in the Instructions for Authors (https://academic.oup.com/plphys/pages/general-instructions) is: Mikael Brosché (mikael.brosche@helsinki.fi).
