# Supplementary material for: Ozone responses in Arabidopsis: beyond stomatal conductance
Source: Plant Physiol. 2021 Feb 24;186(1):180–92. doi: 10.1093/plphys/kiab097 (PMC8154098; doi:10.1093/plphys/kiab097)
Supplement: kiab097_Supplementary_Data [file kiab097_supplementary_data.zip › pp.00132.2021-s08.pdf]

Figure S1.

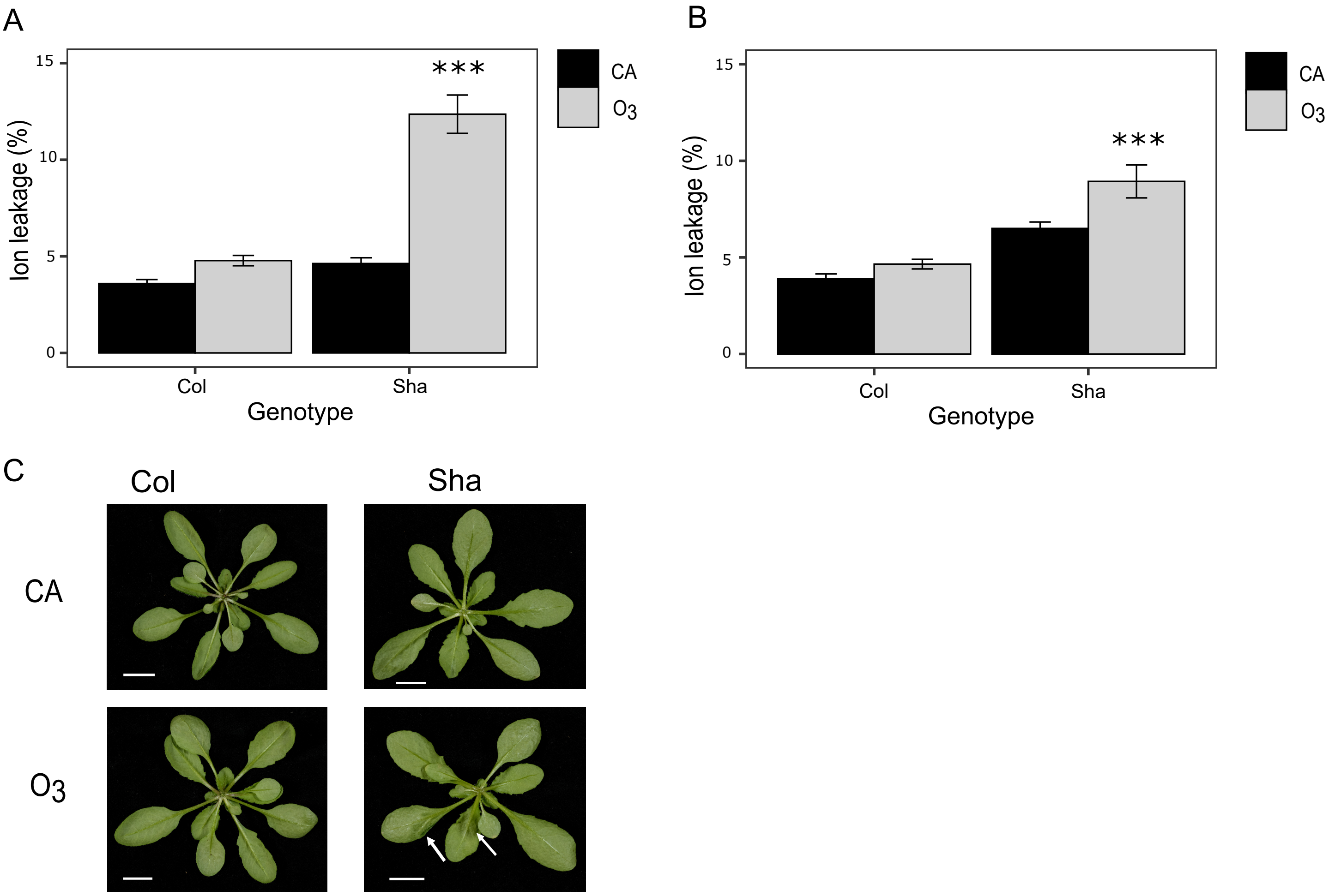

Supplemental Figure S1. O_3 response in Col and Sha plants treated with two different doses of O_3 for 6 h. Cell death measurements with ion leakage in CA control and O_3 treated plants (A) 250 nL L^{-1} and (B) 200 nL L^{-1}. Mean of four independent experiments ± SE is shown (*n = 20*). The asterisks denote significant differences (*P < 0.01*) between Col and Sha assessed with the function fit.contrast from gmodels 2.18.1 (Gregory Warnes et al., 2018). (C) Representative pictures of plants exposed to 250 nL L^{-1} of O_3 taken at the end of O_3 exposure (scale bar 1cm). Arrows indicate O_3-induced lesions in Sha.

Figure S2.

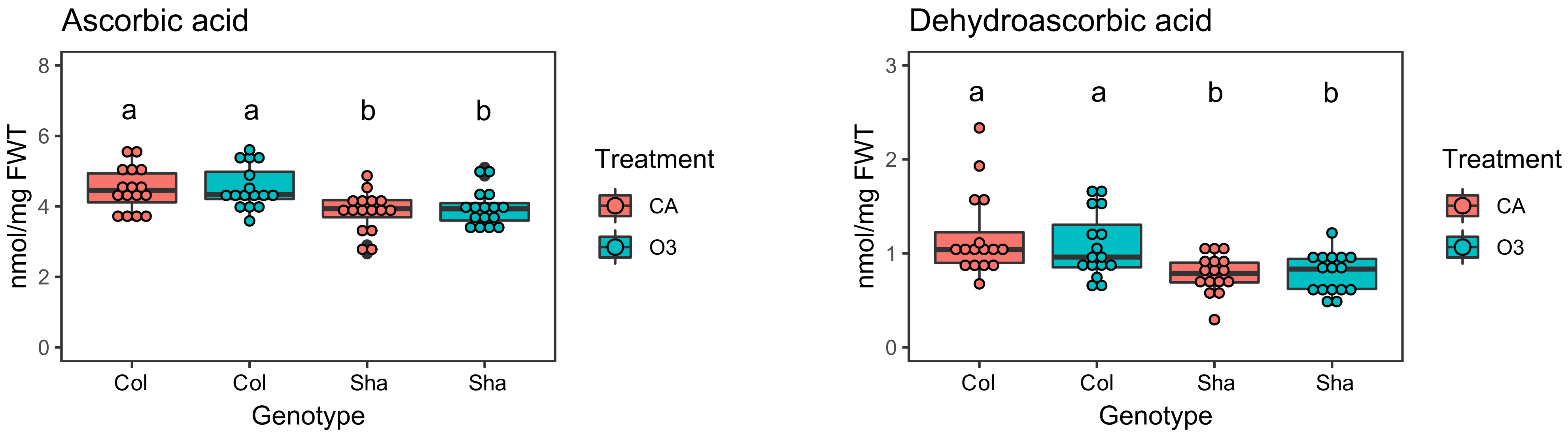

Supplemental Figure S2. Ascorbic acid measurements in Col and Sha plants exposed to 350 nL L^{-1} O_3 for 2 h. Boxplots (center line, median; box limits, upper and lower quartiles; whiskers, 1.5× interquartile range; points, outliers) represent all measurements from four independent experiments with ($n = 16$). Different letters denote significant differences ($P < 0.01$) between Col and Sha assessed with the function fit.contrast from gmodels 2.18.1 (Gregory Warnes et al., 2018). FWT (fresh weight).

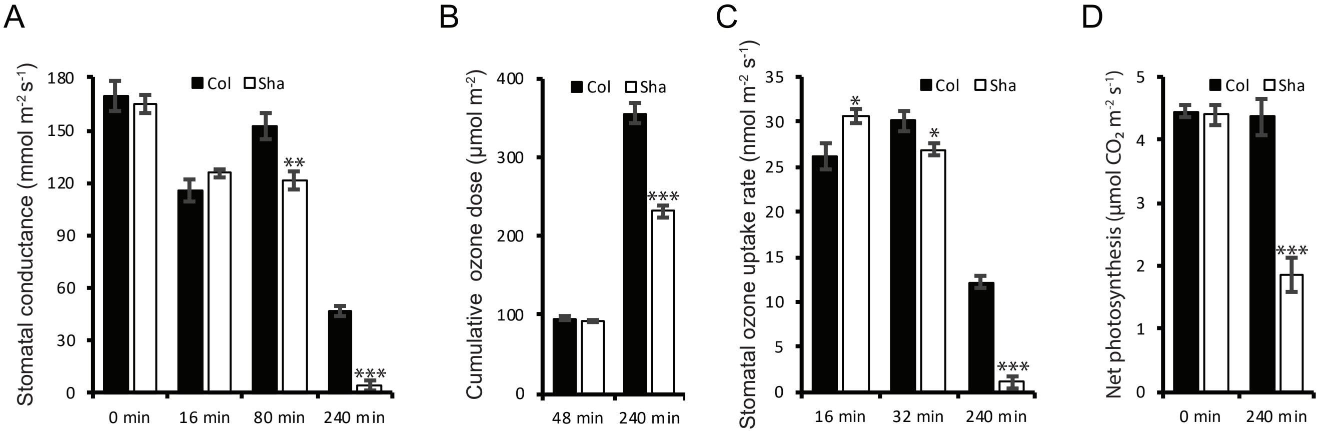

Supplemental Figure S3 . Gas exchange parameters in Col and Sha subjected to ozone treatments. (a) Stomatal conductance, (b) cumulative ozone dose, (c) stomatal ozone uptake rate, and (d) photosynthetic performance are shown at the indicated time points from the data presented in Figure 2. The values are mean±SE (n=12 from 3 independent experiments). Asterisks indicate significant differences between Col and Sha (one-way ANOVA followed by Dunnett's post-hoc test; $P<0.05$; * $p < 0.05$; ** $p < 0.01$; *** $p < 0.001$).

Figure S4.

A. Increased transcript accumulation by O3.   B. Decreased transcript accumulation by O3.

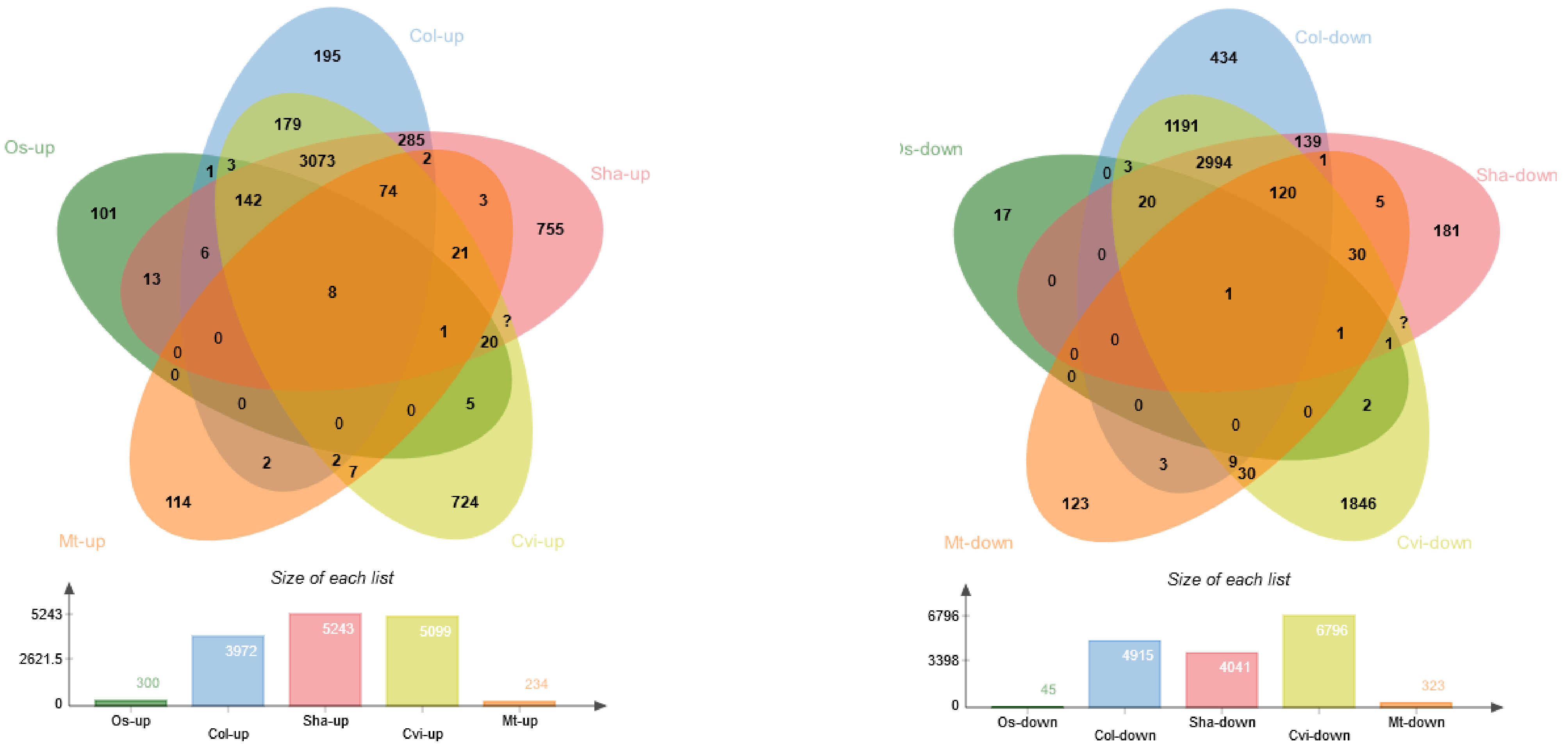

Supplemental Figure S4. Identification of common O_3 regulated genes in Arabidopsis, Medicago and rice. Overlap between genes differentially expressed in Col, Sha and Cvi after the O_3 treatment (350 nL L−1 for 2 h) with Arabidopsis orthologues of genes regulated by O_3 (70 nL L−1, 6 h per day for 6 days) in Medicago (Iyer et al., 2013) and by O_3 (108 nL L^{-1} 7 h per day for 8 days) in rice (Ashrafuzzaman et al., 2018). Genes with increased (A) and decreased (B) transcript accumulation.
